# TLR7 Agonism Accelerates Disease and Causes a Fatal Myeloproliferative Disorder in NZM 2410 Lupus Mice

**DOI:** 10.3389/fimmu.2019.03054

**Published:** 2020-01-10

**Authors:** Jena R. Wirth, Ivan Molano, Phil Ruiz, Sheryl Coutermarsh-Ott, Melissa A. Cunningham

**Affiliations:** ^1^Division of Rheumatology and Immunology, Medical University of South Carolina, Charleston, SC, United States; ^2^Department of Pathology, University of Miami School of Medicine, Miami, FL, United States; ^3^Department of Biomedical Sciences and Pathobiology, Virginia-Maryland College of Veterinary Medicine, Virginia Tech, Blacksburg, VA, United States

**Keywords:** lupus, toll-like receptor (TLR)-7, mouse models, interferon, histiocytosis

## Abstract

Murine models of lupus, both spontaneous and inducible, are valuable instruments to study SLE pathogenesis. Accelerants such as Type I IFN are often used to trigger earlier disease onset. We used a topical TLR7 agonist, previously reported to induce lupus-like disease in WT mice within weeks, to validate this data in C57BL/6j mice, and to test TLR7 agonism as an accelerant in lupus-prone NZM2410 mice. We found that TLR7-stimulated B6 and NZM2410 mice had significantly reduced survival and exhibited profound splenomegaly with significantly reduced B cells (4 vs. 40%), and T cells (8 vs. 31%). Spleen pathology and IHC revealed massive expansion of F4/80+ cells in TLR7-treated mice consistent with histiocytosis. While resiqimod treatment caused mild autoimmunity in B6 mice and accelerated autoimmunity in NZM2410 mice, it did not cause significant nephritis or proteinuria in either strain (renal function intact at death). Given the macrophage expansion, cytopenias, and disruption of normal splenic lymphoid follicle architecture, histiocytic sarcoma is favored as the cause of death. An alternative etiology is a macrophage activation syndrome (MAS)-like syndrome, since the mice also had a transaminitis and histologic hemophagocytosis in the setting of their rapid mortality. For investigators who are focused on murine models of lupus nephritis, this model is not ideal when utilizing B6 mice, however topical resiqimod may prove useful to accelerate autoimmunity and nephritis in NZM2410 mice, or potentially to investigate secondary complications of lupus such as histiocytic diseases or macrophage activation like syndromes.

## Introduction

Systemic lupus erythematosus (SLE) is a chronic autoimmune disease characterized by autoantibody production and immune-complex related end-organ damage. Toll-like receptors (TLRs), especially endosomal TLRs, are implicated in SLE disease pathogenesis, and lupus nephritis in particular (1). TLRs are pattern recognition receptors critical in mediating the innate immune response, responding to the presence of microbial products during infection, for example, and initiating signaling pathways that lead to expression of myriad immune and inflammatory genes (2). TLR7, found in both mice and humans, recognizes single stranded RNA (ssRNA) and activates Type I interferon (IFN) signaling, a pathway active in many lupus patients. In addition to its role in IFN production, TLR7 is thought to play a role in SLE development by activating NFκB-inducible pathways in innate immune cells. The pristane-inducible murine model of lupus, for example, is dependent on TLR7 signaling (3). TLR7 duplication on the Y chromosome (*Yaa* locus) causes male-specific lupus in BXSB mice (4). Likewise, overexpression of TLR7 causes development of lupus, and deficiency of TLR7 in lupus-prone MRL/*lpr* mice ameliorates disease (5–8). Most recently, abnormal maintenance of X inactivation, with TLR7 escape, was implicated in autoimmunity (9, 10). In short, multiple lines of evidence demonstrate that TLR7 dose can increase lupus susceptibility in both mice and humans.

Consistent with this, Yokogawa et al. demonstrated that short-term topical administration of a TLR7/8 agonist to the ears of WT mice induced a lupus-like syndrome, including glomerulonephritis, which was associated with increased IFNα (11). This model required both epicutaneous administration of the TLR7 agonist as well as plasmacytoid dendritic cells. Nephritis and hepatitis were demonstrated in the FVB/N and BALB/c strains, but the extent of nephritis or other lupus-like disease manifestations in treated C57BL/6 mice was less clear. Splenomegaly, which was significant after 4 weeks or 8 weeks of imiquimod cream, administered three times weekly, was the main finding presented in C57BL/6, with the majority of data focused on the other two strains. This inducible model was adopted to study mechanisms of TLR7 action in systemic autoimmunity, and was successfully used to investigate TLR7/TLR9 imbalance in lupus vasculopathy as well as autoimmune-mediated myocarditis (12, 13). TLR7 agonism has the additional benefit of rapid onset of action, and is also being used as an accelerant in spontaneous lupus murine models (14). Herein we utilized this inducible model in WT C57BL/6j (B6) mice to determine if topical TLR7 agonism triggered significant autoimmune-mediated nephritis, since data on this is lacking in the prior publications, and we additionally tested whether it accelerates disease expression in lupus-prone NZM2410 mice.

Although we found that topical TLR7 stimulation caused mild autoimmunity in WT B6 mice as evidenced by low +ANA, no significant nephritis was observed on histology or functional studies (24 h albuminuria), despite high animal mortality. Although we found that TLR7-stimulated NZM2410 mice had both accelerated autoimmunity and nephritis, like B6, treated NZM2410 mice also succumbed from non-nephritis complications. Both strains had profoundly enlarged spleens, profound anemia and thrombocytopenia, and significantly reduced survival compared to vehicle-treated mice. Spleen histology and flow cytometry revealed massive expansion of histiocytes as well as hemophagocytosis, suggesting that repetitive TLR7 stimulation led to death by histiocytosis/histiocytic sarcoma or from a MAS-like inflammatory syndrome.

## Materials and Methods

### Mice

Mice were bred and housed at the Ralph H. Johnson VA animal facility (Charleston, SC, USA). Animal protocols followed the principles outlines in the Guide for the Care and Use of Laboratory Animals and approved by the Ralph H. Johnson VAMC IACUC. The C57BL/J (B6) and NZM2410/J mouse strains were originally acquired from Jackson Laboratory (Bar Harbor, ME, USA). They were maintained on a 12 h light/dark cycle with access to food and water at will. Experimental mice consisted of female and male C57BL/6J mice (*n* = 29, 7–14 weeks) and pre-disease NZM2410 mice (*n* = 31, 13–15 weeks), using littermates when possible.

### R848 Treatment

Mice were treated with the TLR7/8 agonist R848/resiquimod (Enzo Life Sciences, Farmingdale, NY, USA) 100 μg/30 μl in acetone 3× per week on the right ear. Control mice were similarly treated with 30 μl of acetone vehicle. B6 mice were 10–16 weeks old and NZM mice were 13–15 weeks old at treatment start. Mice were dosed for 8 weeks or until they reached sacrifice requirements (>15% of body weight, 3+ proteinuria by dipstick, or upon recommendation by the VA veterinarian).

### Serum Autoantibodies and Urine Protein Excretion

Serum was collected at sacrifice and ANAs were measured by ELISA (NovaTeinBio, Woburn, MA, USA), with an assay sensitivity of 98.9%. Anti-dsDNA levels were assessed by in-house ELISA as previously described (15). Mice were housed in metabolic cages for 24 h urine collection after 3 and 5 weeks of treatment. Antibiotics (ampicillin 25 μg/ml, gentamicin 50 μg/ml, and chloramphenicol 200 μg/ml) were added to the collection tubes. After 24 h, urine volume was measured, and proteinuria was determined by an in-house albumin ELISA using serial-diluted mouse albumin (1 ug/ml) as a known standard.

### Spleen and Bone Marrow Processing

Spleens were harvested and hemisected, half being stored in 10% formalin for histology and half utilized for flow cytometry. Spleen cells were processed on ice through 40 um strainers and depleted of red bloods with red blood cell lysis buffer (144 mM NH_4_Cl and 17 mM Tris, pH 7.6). Cells were washed twice with cold, complete RPMI (10% fetal bovine serum, 1% penicillin-streptomycin, 1% L-glutamine, and 500 μl ampicillin) before being stained for flow cytometry analysis. Bone marrow was harvested from the tibia and femur of both hind limbs. Red blood cells were depleted in the same manner as spleen cells and remaining cells were kept on ice in cold, complete RPMI for flow cytometry staining.

### Cell Staining and Flow Cytometry

Spleen and bone marrow cells (4 × 10^6^/sample) were isolated in staining buffer (1X PBS with 0.5% BSA and 0.02% sodium azide). Viability was assessed using Live/Dead Fixable Dead cell stain (Life Technologies, Carlsbad, CA, USA) at a concentration of 50 μl/million cells in 1X PBS. Cells were washed twice with 1X PBS before panel staining. Panel I staining included F4/80-Brilliant violet 421 (1:100), CD3-Brilliant violet 605 (1:100), CD19-PerCP/Cy5.5 (1:100), pDAC1-APC (1:200), and CD49b-PE (1:300). Panel II staining included CD11c-Brilliant violet 605 (1:100), CD8a-Brilliant violet 421 (1:100), F4/80-Brilliant violet 510 (1:100), Siglec H-PerCP/Cy5.5 (1:100), CD11b-PE (1:300), B220-PE/Cy7 (1:200), and CD3-FITC/CD19-FITC (1:200) as a dump channel. All antibodies were purchased from Biolegend (San Diego, CA, USA). Cells were incubated with antibodies for 30 min on ice in the dark. Cells were washed twice with staining buffer and suspended in 0.3 ml staining buffer for flow cytometry. Cells were acquired on an LSR Fortessa X20 cell analyzer (BD Biosciences, San Jose, CA, USA) and analysis was performed using FlowJo software (FlowJo LLC, Ashland, OR, USA).

### Spleen and Kidney Histology

Hemisected spleens were stored in 10% formalin until processed for histology. Spleen tissues samples were stained with H&E and Periodic acid-Schiff (PAS) stains and analyzed blindly by a veterinary pathologist with expertise in spleen assessments. Immunohistochemistry was also performed on spleen sections to confirm histiocytes with an F4/80 antibody using a red chromogen and DAB counterstain. Kidneys were also stored in formalin until the tissue was prepared for histology and stained with H&E. Renal slides were read and interpreted in a masked fashion by a clinical pathologist who graded the kidneys for glomerular hypercellularity, segmental mesangial expansion, neutrophils/cell debris, crescent formation, and necrosis. These parameters were combined for a total glomerular pathology score with a maximum score of 20. Interstitial changes and vasculitis were also noted and graded separately. Scores from 0 to 4 were assigned to each of the features and added together to yield a final renal score.

### Blood Counts and Metabolic Analysis

Blood was collected in lithium heparin BD Microtainer (BD, Franklin Lakes, NJ) tubes at the time of sacrifice via cardiac puncture and was analyzed by the MUSC Division of Laboratory Animal Resources. Albumin, alanine aminotransferase, blood urea nitrogen, and creatinine were measured via the VetScan V2 Chemistry Analyzer Comprehensive Diagnostic Profile rotor (Abaxis, Inc., Union City, CA). White blood cell count, hematocrit, and platelet count was assessed on a Hemavet 950 (Drew Scientific Group, Miami Lakes, FL).

### Statistics

Logrank analysis was used to compare trends in animal survival. Two-way ANOVA was utilized to test for significance when comparing groups in autoantibody, proteinuria, and flow cytometry assays. Spearman correlation coefficients were calculated for IFNα and albumin correlations with mortality. Mann–Whitney non-parametric *t*-tests were utilized to test for significance between groups in single group comparisons as in pathology scoring. Standard error of the means was reported where applicable. *p* ≤ 0.05 were considered significant. Mice within treatment groups died at different timepoints, thus final weights of all mice were carried forward to end of treatment (8 weeks).

## Results

### Topical Treatment With a TLR7 Agonist Resulted in Splenomegaly and Early Mortality

**Figure 1:**
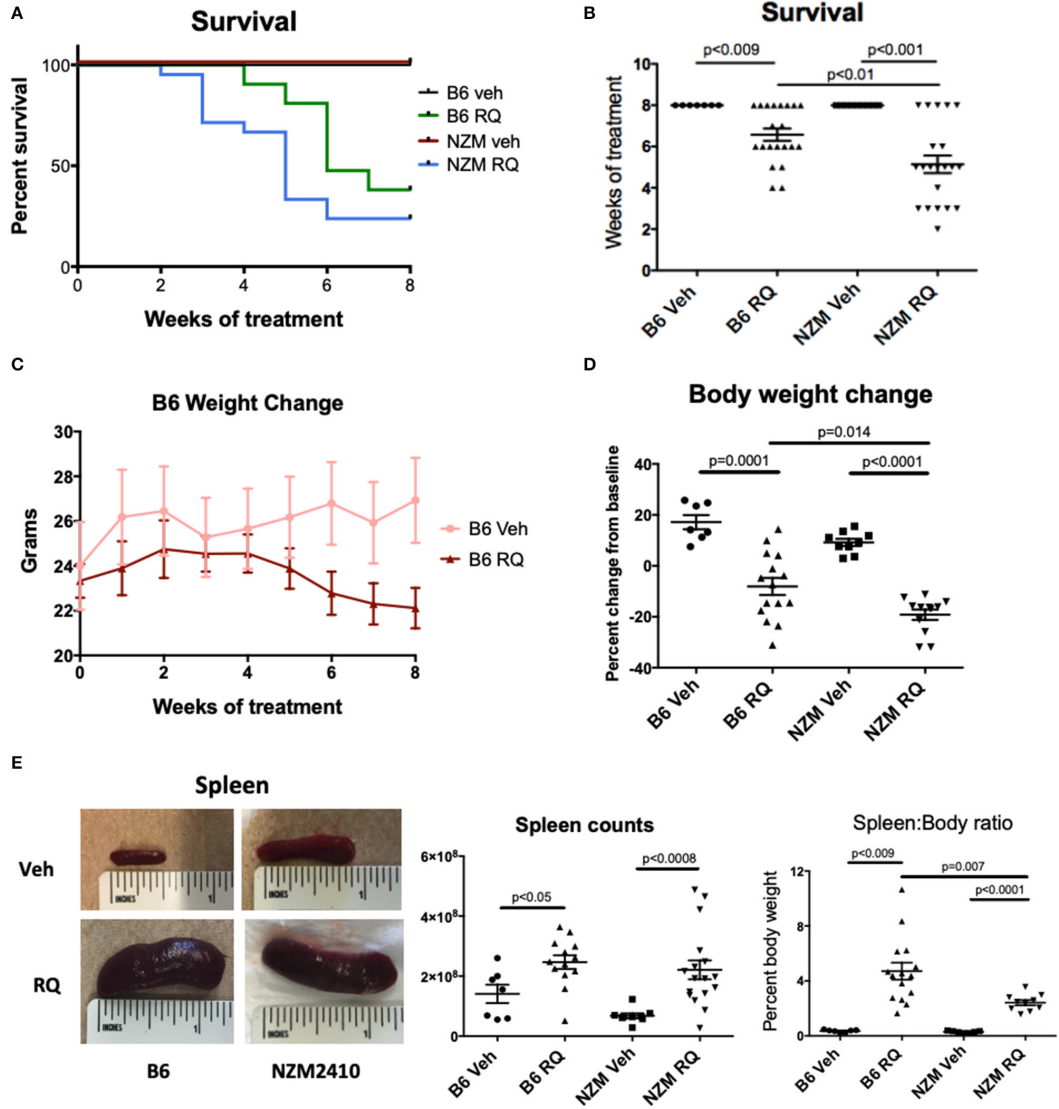
Survival of WT C57BL/6 and NZM2410 mice after treatment with a topical TLR7/8 agonist (R848, RQ). **(A,B)** TLR7/8 stimulation in both B6 and NZM mice resulted in significantly reduced survival. Median time to death in TLR7-stimulated NZM mice was also significantly accelerated vs. treated B6 WT mice. **(C,D)** TLR7-treated mice lost significant weight and **(E)** spleens were profoundly enlarged, with significant increases in spleen cell counts.

Topical TLR7 agonist treatment with R848 (resiquimod) significantly decreased survival in both WT B6 and lupus-prone NZM2410 mice compared to the acetone control groups. In contrast to previous reports, decline in survival was rapid in both groups, starting at 4 weeks after treatment in B6 mice, and 2–3 weeks in NZM mice. In fact, <50% of treated mice survived beyond 6 weeks of treatment, while all control mice survived to 8 weeks, the pre-determined endpoint at which time all mice were sacrificed (Figures 1A,B). Body weights of B6 mice were similar at treatment start, however the TLR7**-**stimulated group did not gain weight normally and subsequently lost weight as treatment continued and the mice became moribund (Figure 1C). TLR7-stimulated pre-disease NZM mice also lost significant body weight (Figure 1D). Both strains of treated mice had significant splenomegaly compared to control mice (Figure 1E). The extent of splenomegaly did not correlate with survival in treated B6 mice; for example, the four B6 mice with the highest (>6%) spleen:body ratio all completed the full 8-weeks course of treatment. Interestingly, TLR7-treated female mice trended toward earlier death vs. males (67% of female B6 dead by 6 weeks vs. 30% males; 46% female NZM dead by 4 weeks vs. 12.5% males), but the overall difference did not meet significance.

### Topical TLR7 Agonist Treatment Increased Serum Autoantibody Production and Serum IFNα

**Figure 2:**
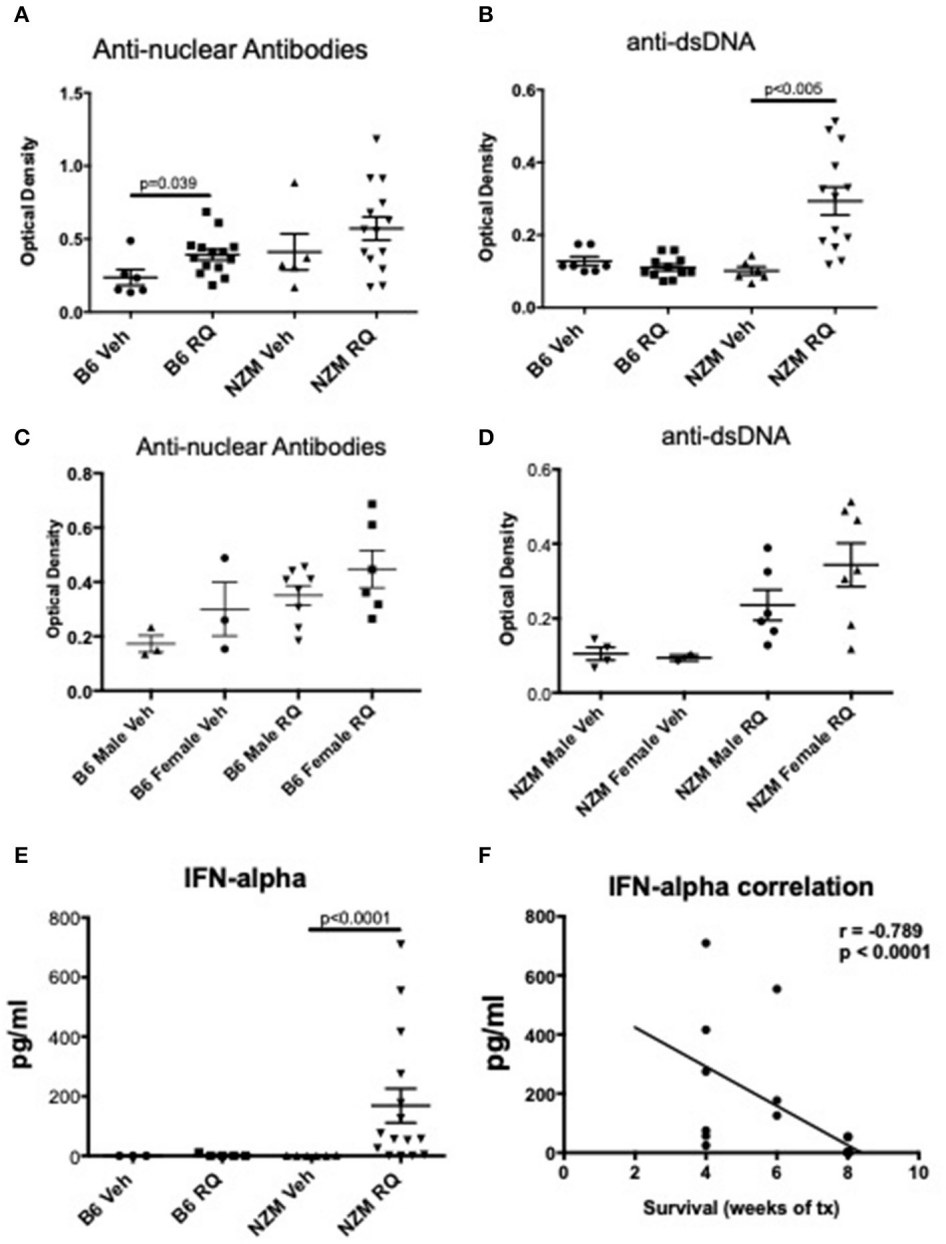
Autoimmunity and IFNα levels in TLR7-stimulated WT and NZM2410 mice. **(A,B)** We observed low +ANAs, but not anti-dsDNA Abs, in TLR7-stimulated B6 mice. NZM mice had both +ANAs and +anti-dsDNA Abs, as would be expected for age (16–23 weeks); serum levels of anti-dsDNA were significantly increased in TLR7-stimulated mice. **(C,D)** Female B6 and NZMs trended toward higher ANAs and anti-dsDNA Abs, respectively. **(E,F)** IFNα levels were also significantly increased in NZM mice and correlated with earlier death.

To assess induction of autoimmunity after TLR7 stimulation, ANAs were analyzed by ELISA. Treated B6 mice had higher ANA titers (*p* = 0.039), but not anti-dsDNA antibody levels, while treated NZM mice trended toward higher levels of ANA, and had significantly higher anti-dsDNA levels (*p* < 0.005) (Figures 2A,B). As might be expected with TLR7 stimulation, female mice trended toward increased autoantibody production compared with male mice (Figures 2C,D). Serum IFNα levels were also measured by ELISA in a subset of mice. By this measure, TLR7-stimulated NZM mice, but not B6, had significantly increased serum IFNα levels compared with vehicle-treated littermates, *p* < 0.0001 (Figure 2E). Additionally, IFNα levels were significantly inversely correlated with survival (i.e., the higher the serum IFNα level, the earlier the death) (Figure 2F). Serum IFNα levels were not higher in female vs. male NZM mice treated with topical resiquimod (data not shown).

### TLR7 Stimulation Accelerates Glomerulonephritis in NZM2410 Mice

**Figure 3:**
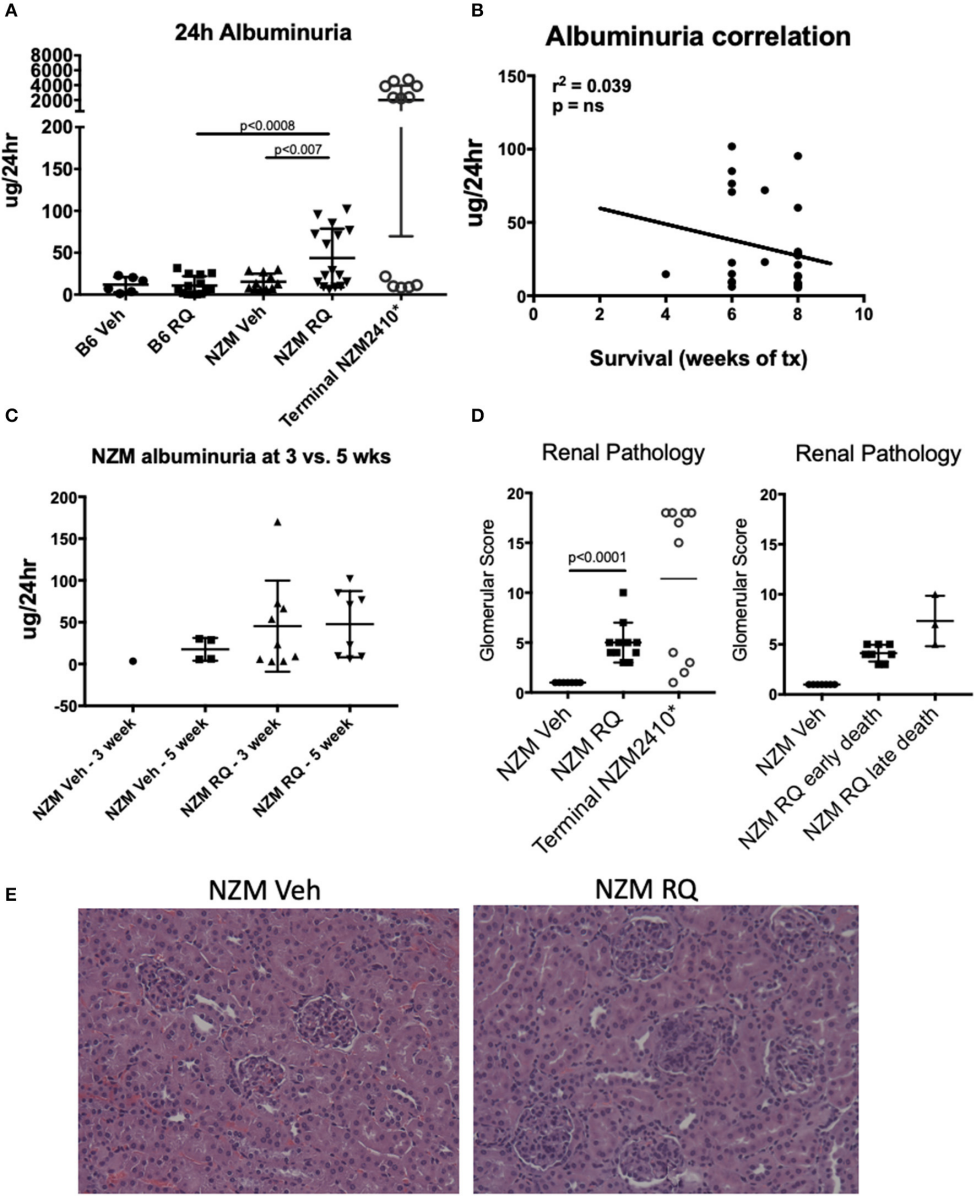
Despite accelerated death after TLR7/8 agonism, both B6 and NZM mice had minimal proteinuria. **(A–C)** TLR7-treated NZM mice had significantly increased 24 h albuminuria vs. vehicle-treated NZM and TLR7-treated B6, however, these levels were not elevated to the degree typically seen in lupus prone mice that succumb from renal failure (*terminal 24 h proteinuria in NZM2410 mice is usually >1–2 mg); albuminuria also did not correlate with earlier death. **(D,E)** Renal pathology demonstrated significantly higher glomerular scores (GS) for nephritis (proliferative lesions, mesangial expansion, etc.) in TLR7-treated NZM, but not to the degree seen in terminally nephritic mice (GS typically ~13–18), and mice that died earlier had milder nephritis.

Urine protein levels (24-h collection) were assessed via albumin ELISA after 5 weeks of treatment (and at an additional earlier 3-weeks timepoint in a subset of mice). We and others showed in previous studies that NZM2410 mice develop clinically apparent lupus nephritis starting at ~18 weeks of age, with subsequent progression to end stage disease renal disease and death (~50% dead by 26–30 weeks). This is true for most, but not all mice, as some escape progression and recover (15–17). We started topical resiquimod treatment of NZM2410 mice at 10 weeks of age, and assessed protein levels at 15 weeks of age, allowing for observation of possible early onset of renal disease due to TLR7 agonism. In B6 wild-type mice, minimal/trace proteinuria was observed in either TLR7-stimulated or vehicle-treated mice (Figure 3A). TLR7-stimulated NZM2410 mice demonstrated significantly increased proteinuria compared to both control NZM2410 mice (as well as TLR7-stimulated B6 mice). However, proteinuria, despite being measured at the time of death for some animals, were minimal (avg 44 μg/24 h) compared to levels typically observed in (older) nephrotic NZM2410 mice at time of death (Figure 3A, avg 2,000 μg/24 h). Albuminuria in these 15 weeks old TLR7-stimulated NZM2410 mice were similar to that seen in NZM2410 mice at 18 weeks of age (early stage renal disease, typically 0–100 μg/24 h) (18), so albuminuria is indeed accelerated for age in comparison to vehicle-treated NZM mice. This level of albuminuria would not be expected to cause death from renal failure. Additionally, albuminuria was not correlated with mortality (Figure 3B), and no significant difference was seen between 3 and 5 weeks after treatment (Figure 3C), suggesting this low level of proteinuria is enhanced in TLR7-stimulated lupus-prone mice, but is not rapidly progressive, nor the cause of death in the young TLR7- stimulated mice. Lastly, renal histopathology on NZM2410 kidneys also suggested accelerated nephritis in the TLR-stimulated group (glomerular score = 5, vs. 1, *p* < 0.0001) (Figures 3D,E), but again not to the extent that we typically see in NZM2410 mice with end stage nephritis/nephrosis (terminal glomerular scores usually >14) (18, 19). Death from a non-nephritis cause is further indicated by NZM2410 mice that died early (4–6 weeks post-treatment vs. 8 weeks). Mice that died earlier had *less* severe renal histology scores, although this did not meet significance. These findings suggest the trend in pathology scores of resiquimod treated NZM mice was likely age-related (Figure 3D). Thus, while TLR7 stimulation is accelerating kidney disease in NZM2410 mice, the kidney disease observed, as measured by both histopathology and a functional endpoint, did not result in their death. It follows that TLR7-stimulated B6 mice, which had minimal/trace proteinuria, also died from a non-nephritis related cause.

### Hematologic Derangements in TLR7-Stimulated NZM2410 Mice

**Figure 4:**
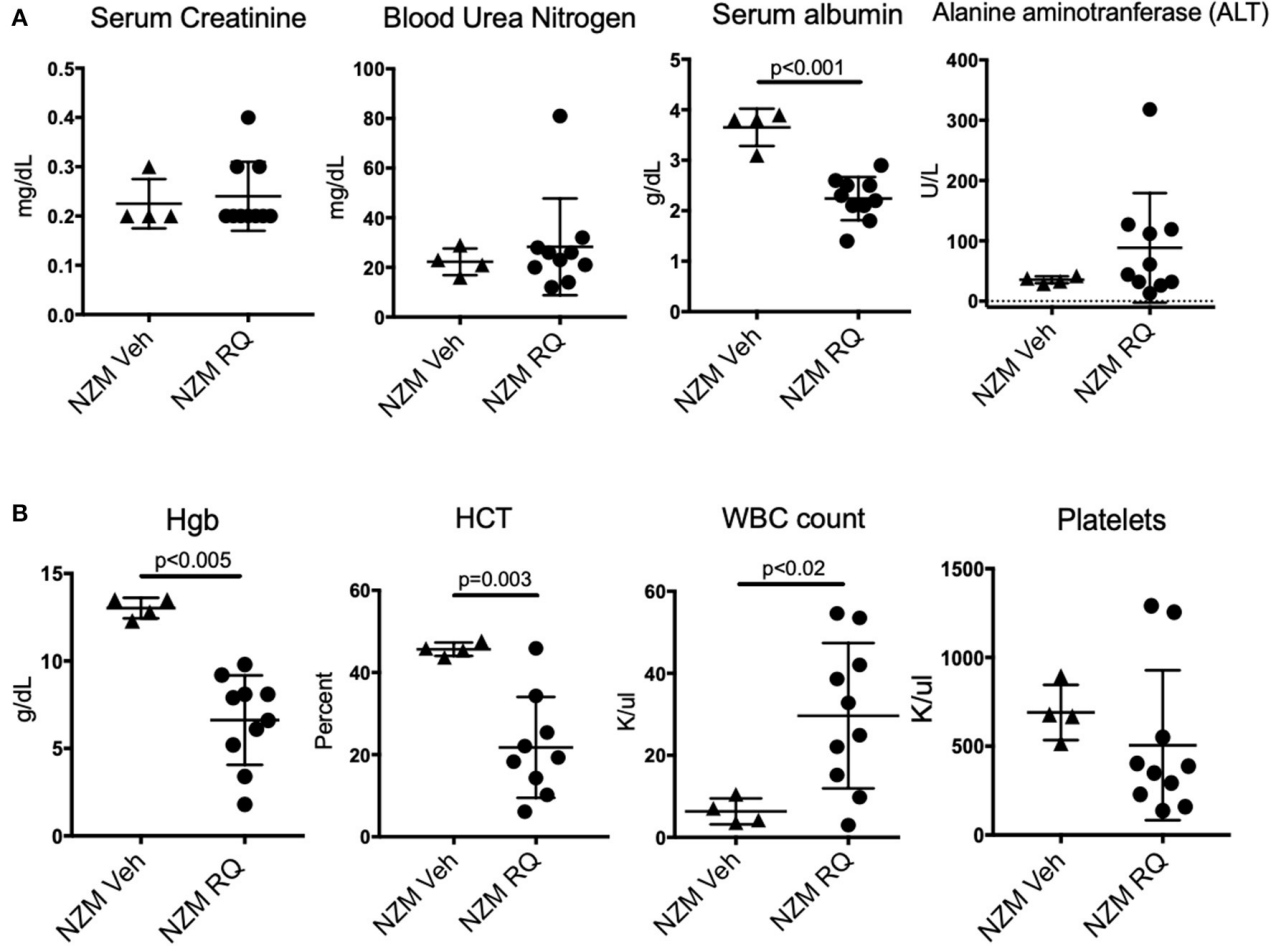
TLR7-stimulated NZM2410 mice have normal serum BUN and creatinine, but have hematologic derangements. **(A)** We observed normal metabolic panels in TLR7-treated mice, except for significant hypoalbuminemia, and transaminitis in a subset of TLR7-treated mice. **(B)** TLR7-treated NZM mice were severely anemic, and had a leukocytosis, more consistent with malignancy, or chronic infection than lupus nephritis/ESRD (above). Most TLR-7 treated mice were also thrombocytopenic.

Complete blood counts (CBC) and complete metabolic panels (CMP) were done in a subset of NZM2410 mice. Consistent with the minimal albuminuria and only mild histopathologic changes seen in kidneys from treated mice, serum creatinine was preserved in the normal range in TLR7-stimulated mice, regardless of age at death (Figure 4A). We also did not observe changes in blood urea nitrogen (BUN), a second endpoint for renal function. Serum albumin, however, was significantly reduced in TLR7-stimulated mice, likely due to their ongoing low-grade proteinuria, as well as the moribund state of these mice at the endpoint of the study. Liver enzymes, as reflected by alanine aminotransferase (ALT), trended upward in TLR7-stimulated NZM2410 mice, but was not significantly increased. More profound changes were seen in their blood counts, as TLR7-stimulated mice had statistically and clinically significant anemia as well as a leukocytosis (Figure 4B). The WBC counts were more than 5-fold increased in some animals, suggesting a possible myeloproliferative process vs. infection. The severity of the hypoalbuminemia and anemia further suggest the possibility of a histiocytic disease in these mice. TLR7-stimulated mice also tended to have thrombocytopenia (~80% of mice), however, this was not overall significant since two resiquimod-treated NZM2410 mice had a thrombocytosis.

### Effects on CD19+ and CD3+ Splenocytes and Bone Marrow Cells

**Figure 5:**
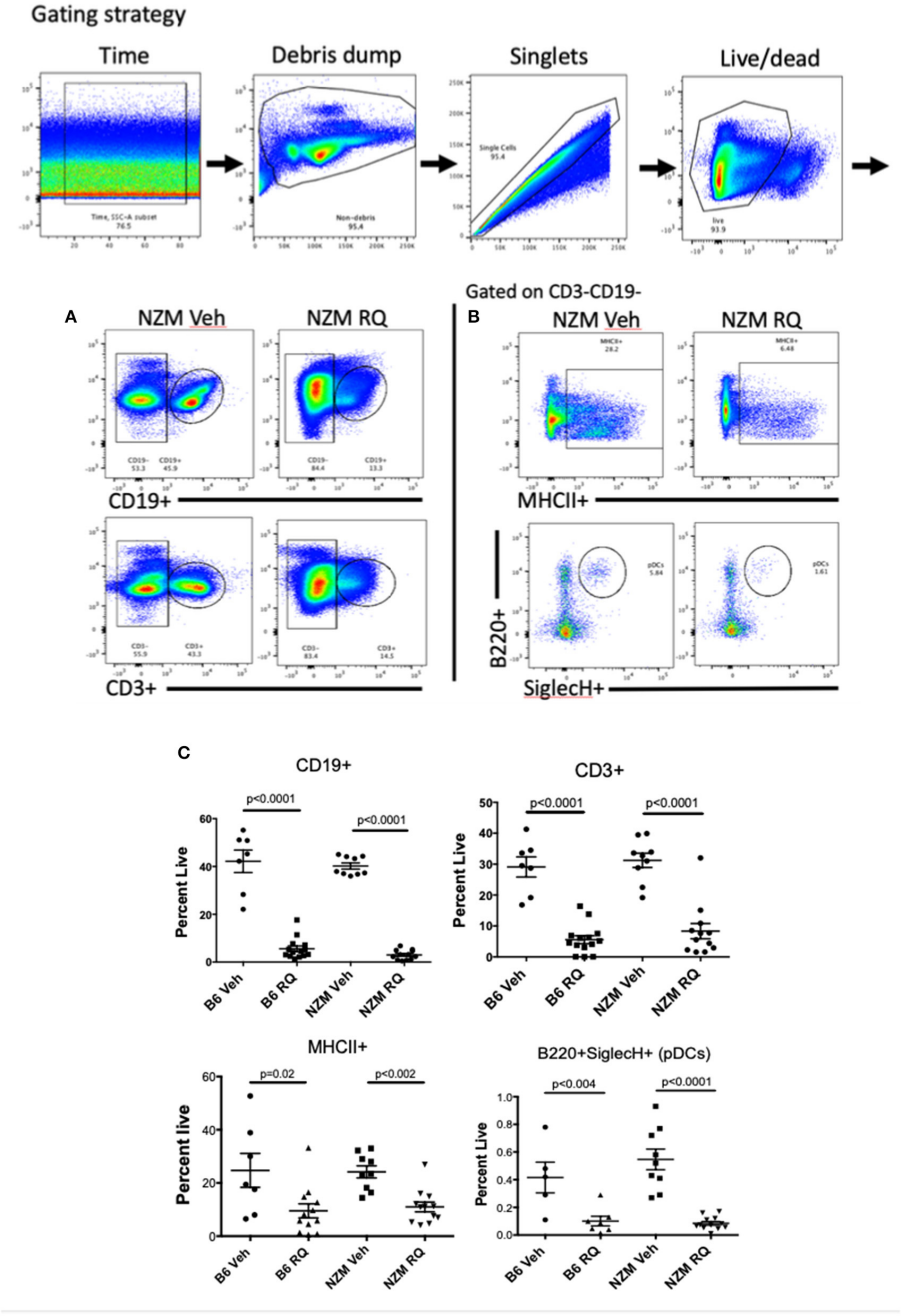
Spleen immunophenotype of topical TLR7-treated B6 and NZM mice. Flow cytometry revealed profound decreases in **(A,C)** CD19+ B cells and CD3+ T cells as well as **(B,C)** MHCII+ cells and pDCs from spleens from TLR7-stimulated B6 and NZM mice. Similar decreases in B cell counts, MHCII+ cells and pDCs were seen in bone marrow (BM) from these animals, while CD11b+ cells were significantly increased in BM (Supplemental Figure 2).

General markers of B and T cells (CD19+ and CD3+) were used to assess the impact of topical TLR7-stimulation on adaptive immune cells in the spleen. Surprisingly, the percent of B and T cells were both significantly decreased in TLR7-stimulated mice, regardless of strain (Figures 5A,C); absolute numbers were also significantly decreased (Supplemental Figure 1), despite significant splenomegaly in TLR-stimulated mice. We also examined MHC II positivity, after gating on CD19-CD3- cells, as a general assessment of antigen presenting cells (APCs) present, which we expected might be increased following TLR stimulation. MHC positivity, however, was decreased in both strains of TLR7-treated mice (Figures 5B,C). NK Cells (CD49+) (data not shown) and plasmacytoid DCs (B220+SiglecH+) (Figures 5B,C) were also significantly decreased in the spleen. Unexpectedly, the MHCII+CD11c+ population was not significantly changed, but CD11c+CD11b+ and MHCII+CD11b+ cell populations were significantly increased in spleens from TLR-stimulated mice (Supplemental Figure 1). In general, cDC2 cells (MHCII+CD11c+CD11b+) were found at a much higher frequency than cDC1 cells (MHCII+CD11c+CD11b-CD8a+) which were significantly reduced (Supplemental Figure 1). Lastly, the total number of CD11b+ cells in the spleen was decreased, likely due to the decreased number of B cells which may express CD11b.

*Ex vivo* bone marrow cells were also analyzed by flow cytometry. TLR-stimulated mice had a significantly decreased percent of B cells (CD19+) as well as macrophage (F4/80+) and pDC (B220+SiglecH+) (Supplemental Figure 2). There was a trend toward increased CD11c+ cells in BM, but surprisingly, not the massive expansion that was seen in prior studies with TLR7 transgenic overexpression. Unlike the spleen, CD11b+ and CD11b^hi^ cells were significantly increased in TLR7-stimulated mice compared to the control mice, but were not MHCII+ (Supplemental Figure 2B), suggesting they are not mature/activated macrophage, but perhaps monocytes. Consistent with this, F4/80+ cells were actually reduced in BM as well. Together these data suggest that the spleen histiocytes that are expanded in resiqimod treated mice are monocyte-derived macrophages as was seen in TLR7 transgenic mice (20). There may also be an element of bone marrow (BM) failure contributing to the TLR7-induced phenotype (which lends itself to a diagnosis of a myelodysplastic syndrome). BM cell counts trended downward in treated mice, although there was no significant difference in BM cell counts between vehicle and resiquimod treatment within strains (Supplemental Figure 2A).

### Topical TLR7 Stimulation Is Associated With Marked Proliferation of F4/80+ Mononuclear Cells

**Figure 6:**
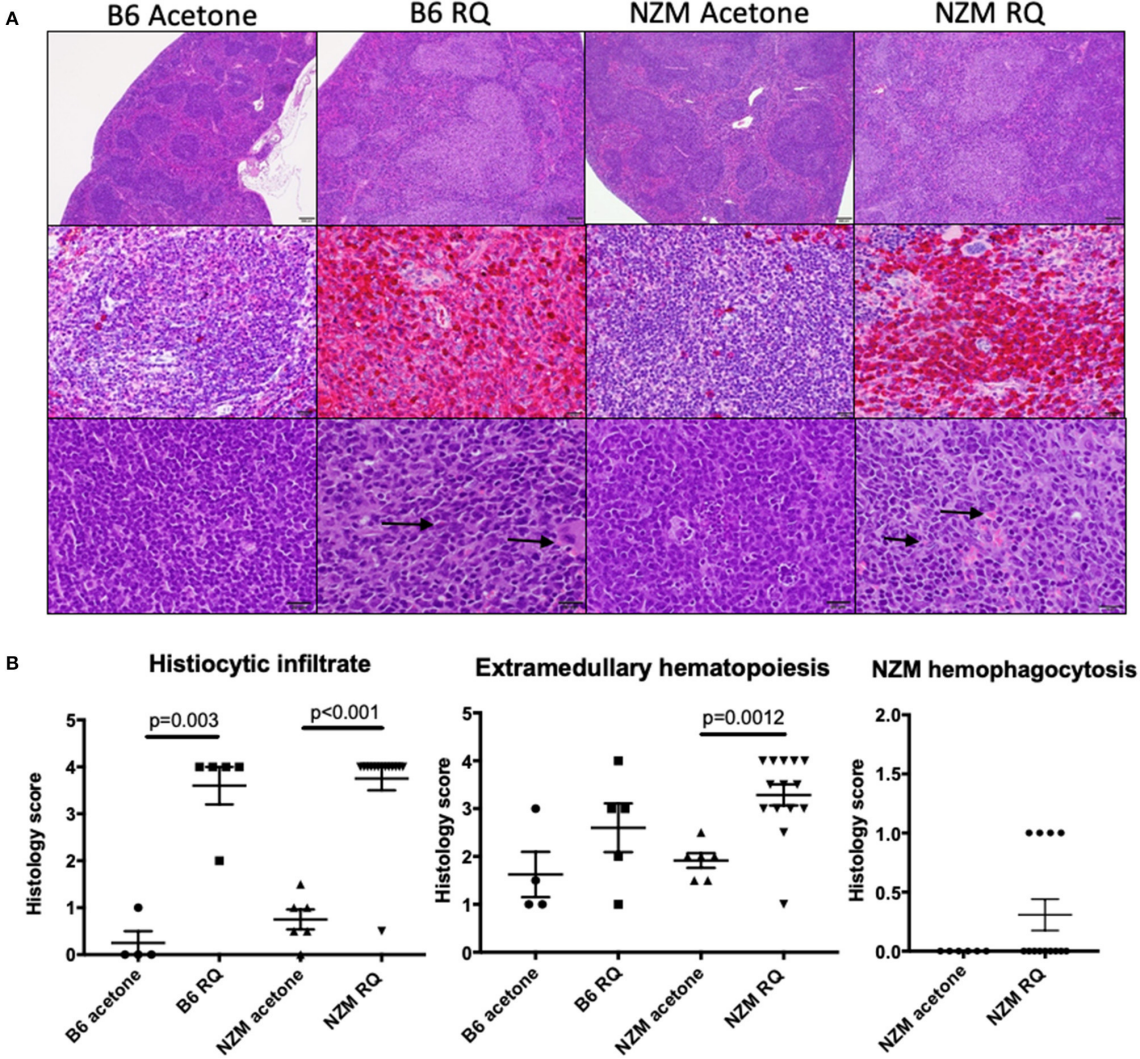
Topical treatment with TLR7/8 agonist is associated with marked proliferation of mononuclear cells in the spleen, consistent with histiocytic sarcoma. **(A)** In vehicle-treated mice, the normal lymphoid follicular architecture of the spleen is maintained whether B6 or NZM (top, low magnification) and only rare, scattered cells within the lymphoid follicles exhibit strong staining for F4/80, consistent with histiocytes/macrophages (middle). In TLR7(RQ)-stimulated mice, there is effacement of the normal lymphoid follicles by mononuclear cells (top, low magnification). In these mice, 80–90% of spleen cells exhibit strong staining with F4/80 (middle) consistent with histiocytic cells. These cells frequently exhibit hemophagocytosis (bottom image, arrows). The top and bottom images are stained with H&E. The middle images are stained with an F4/80 antibody using a red chromogen and DAB counterstain. **(B)** Semi-quantitation of spleen histiocytic infiltrate, extramedullary hematopoiesis, and hemophagocytosis between animal groups.

As demonstrated in Figure 1, resiqimod treated B6 and NZM2410 mice develop marked splenomegaly. Analysis of spleen cell subsets by flow cytometry revealed a significant reduction in B and T cells, as well as in other mature cell types (MHCII+ pDCs). Of the CD11c+ cells, the significant majority were CD11b+, suggesting an increase in cells from the monocyte/macrophage lineage. We then did histologic analysis of spleens from the treatment groups. In vehicle-treated mice, the normal lymphoid follicular architecture of the spleen was maintained whether B6 or NZM2410. In TLR7-stimulated mice, however, there was effacement of the normal lymphoid follicles by mononuclear cells (Figure 6A). Spleen sections were then stained for F4/80 expression. In spleens from vehicle-treated mice, only rare scattered cells, within the lymphoid follicles, showed strong staining for F4/80, consistent with histiocytes/macrophages. In contrast, 80–90% of spleen cells from TLR-stimulated mice exhibited strong staining for F4/80 consistent with histiocytic cells. Spleen histology scores demonstrated a significant increase in histiocytic infiltrate and extramedullary hematopoiesis in TLR7-stimulated mice (Figure 6B). Importantly, the effacement of white pulp and overall loss of normal lymphoid follicular architecture suggested a myeloproliferative neoplastic process in TLR7- stimulated mice, consistent with their cytopenias, weight loss, and significantly reduced survival (without obvious other organ failure). While TLR7-stimulated lupus-prone mice demonstrated mild-moderate acceleration of autoimmunity, Type I IFN production and lupus nephritis, there was no change in renal function at the time of death. Thus, early mortality was most likely the result of TLR7-induced myeloproliferative disease, likely a histiocytic proliferative syndrome or a histiocytic sarcoma, although given the observed hemophagocytosis and high mortality, a macrophage activating syndrome like disease cannot be ruled out.

## Discussion

TLRs are known drivers of autoimmune responses by stimulating innate immune cells and self-reactive B and T cells when exposed to endogenous nucleic acids (7, 21, 22). Endosomal TLRs, TLR 7 in particular, is implicated in lupus disease pathogenesis in mice. TLR7 deletion protects against anti-RNA B cell responses and duplication of TLR7 alone results in autoreactivity and pathology (7, 8, 21). Genetics of human lupus disease also support a role for TLR7 (23). TLR7 agonists have long been utilized in pre-clinical studies as immune adjuvants that enhance anti-tumor and anti-viral immunity. In many of these studies TLR7 agonists are variable in their potency and administration (intra-tumoral, intra-muscular and intra-peritoneal injections as well as intranasal and epicutaneous/topical administration). R848 (resiquimod) and the structurally related compound imiquimod are the most common ligands for TLR7/8 utilized. These imidazoquinolines are potent immune response modifiers, even when applied topically to the skin, which is a key immune location for triggering of lupus disease activity. Thus, it is quite rational to consider this epicutaneous TLR7-inducible phenotype, which is pDC-dependent and partially IFN-dependent, as a viable lupus animal model (11, 14).

The goal of the present study was to investigate and validate topical TLR7 stimulation as an inducible murine model of lupus-like disease in C57BL/6j (B6) mice, as well as an accelerant in NZM2410 lupus-prone mice. Although B6 mice were among the WT strains utilized in the original study by Yokogawa et al., there was much less phenotypic data presented regarding effects in the B6 strain (vs. BALB/c and FVN/B) (11). Nephritis and hepatitis were demonstrated in the FVB/N and BALB/c strains, but the extent of nephritis or other lupus-like disease manifestations in treated C57BL/6 mice was not shown. Splenomegaly, which was significant after 4 or 8 weeks of imiquimod cream administered three times weekly, was the main result presented from B6 mice, while the majority of data focused on the other 2 strains. We felt it was important to extend and validate this model in B6 mice, since they are the most widely used inbred strain and among the most commonly used to generate congenic mice (24). This TLR7-inducible model was first published in 2014 and has since gained popularity because disease induction is rapid (shorter than pristane-induced lupus-like disease, which is also TLR7-mediated), and the lupus phenotype is principally dependent on pDCs/Type I IFNs, which are of import in human disease. Understanding TLR7 as a mediator of lupus disease expression is also important for those studying sex bias in autoimmunity, since females are pre-disposed to TLR7-driven autoimmunity, and aberrant X chromosome inactivation was implicated in increased X-linked TLR7 gene dosage (25).

Herein, we found that topical resiquimod treatment accelerated autoimmunity and lupus disease activity in both male and female NZM2410 mice. B6 mice developed only mild autoimmunity. The B6 mice had low positive ANA titers, but no lupus-like renal disease, and thus would not be an optimal WT strain for use as a TLR7-induced lupus model. One limitation to this study is that we did not examine anti-RNA autoantibody production, which is impacted by TLR7 gene dosage in mice (7, 8). We also did not attempt to reproduce this model in FVB/N or BALB/c mice, which, with resiquimod treatment developed significant glomerular immune complex deposition, elevated serum creatinine and nephritis on pathologic exam. This is in contrast to what we observed in B6 and NZM2410 mice that died with renal function intact (no proteinuria and/or normal serum creatinine).

Unexpectedly, topical TLR7 treatment caused a severe histiocytosis, hemophagocytosis, and hematologic derangements that resulted in high mortality in both strains of mice. Most animals succumbed quickly (within 6 weeks). Similar to what was previously reported in FVB/N and BALB/c, we observed significant mortality in TLR7-stimulated mice that was strikingly swift (the first NZM2410 mouse died within 2 weeks, with mean survival of 5 weeks; 6.5 weeks for B6). Although there was a trend toward earlier death in females, there was not statistically significant sexual dimorphism. Also consistent was the profound splenomegaly seen in resiqimod treated mice. Flow cytometry and pathology revealed the splenomegaly was secondary to increased cellularity (not congestion), and with significant reduction in B and T cells with concomitant increase in F4/80+ cells (shown most convincingly by IHC). We observed a significant reduction in total number of splenic B and T cells in resiquimod treated mice, whereas Yokogawa et al. reported an increase in both lymphoid and myeloid cells, as well as increased activation markers (ex. CD69) on B and T cells. Although it was initially surprising to observe this stark difference between the strains used in Yokogawa's work and in the strains we used, we think the results are complementary, and a function of not only different mouse backgrounds and different mouse colonies, but the timing and potency of different TLR7 agonists. Yokogawa et al. used both imiquimod and resiquimod (R848) in their study, and reported their imiquimod (rather than R848) results at 8 weeks of treatment. R848 is considerably more potent and extended use likely caused the rapid expansion of these myeloid cells that led to mortality in our mice. Potency differences may also be due to a difference in absorption between cream as the conduit versus acetone, which is a weak irritant and more penetrant (acetone was used in this study). Thus, lymphoid expansion and activation likely occurred earlier in our mice and was subsequently superseded by the continued myeloid expansion. The observed reduction in lymphoid cells from bone marrow was also likely the result of the myeloid expansion (similar to what is seen in human myelodysplastic diseases). This is actually supported by data in the Yokogawa paper, demonstrating that R848-treated mice had more severe organ inflammation, as well as much larger spleens with spleen histology showing disrupted follicles. Yokogawa did not show flow cytometry of B or T cells from spleens of these mice, but the histology suggests that it would be similar to what we observed.

Strain-specific differences in TLR7-inducible effects may also have contributed to differences seen in the current work versus the original study. It is worth noting that both B6 and NZM2410 mice had near-identical phenotypes with regard to myeloproliferation and spleen pathology. It is certainly the case that WT strains of mice are variable in their propensity for induction of autoimmunity and related disease manifestations. B6.129 mice, for example, are closely related to C57BL/6 (B6) but have an increased propensity for autoimmunity. Also, of note, BALB/c mice are routinely used for pristane-induced lupus, but other WT mice, such as B6, do not respond to pristane in the same way. Since the pristane model is TLR7-dependent, it is likely that BALB/c mice have a genetic susceptibility to TLR7 induced autoimmune disease that is not present in B6 mice.

The dramatic effect of high doses of TLR7 agonists (also seen in TLR7.tg strains) is to more specifically stimulate the myeloid compartment, which express higher levels of TLR7. An important difference in our study with regard to myeloid expansion is that we observed an increase in CD11b+ in BM, and F4/80+ cells in spleen, but not CD11c+MHCII+ cells, which is consistent with monocyte-derived macrophages, rather than dendritic cell expansion as was reported in TLR7.tg mice (22). In TLR7.tg mice, a small increase in TLR7 expression results in autoreactive lymphocytes, but a large increase in TLR7 quickly results in fatal inflammatory pathology with profound myeloid dysregulation/expansion (7). The latter findings are consistent with our results with the exception that we did not see the same degree of proliferative glomerular disease. Instead, B6 mice had no renal disease, and NZM2410 mice had minimally accelerated nephritis. Strong evidence that resiquimod treatment resulted in death by unchecked myeloproliferative disease, rather than nephritis, was the lack of severe proteinuria and the preserved renal function by BUN and serum creatinine at the time of death. Instead, resiquimod treated mice had significant hematologic derangements—particularly anemia and thrombocytopenia. We also observed significant extramedullary hematopoiesis and hemophagocytosis in spleens of resiquimod treated NZM2410 mice, consistent with possible inflammatory hemophagocytes (iHPCs) and macrophage activation syndrome (MAS)-like disease, as observed in similar chronic TLR7-driven models (20). In the setting of chronically increased TLR7 signaling, a specialized monocyte-derived macrophage phenotype develops, preferentially promoted, and enriched for genes that distinguish these macrophage from other tissue-resident macrophage; the hallmark function is hemophagocytosis (20).

In summary, we utilized the Yokogawa protocol for epicutaneous TLR7 stimulation to induce a lupus-like phenotype and observed mild autoimmunity in B6 mice and accelerated autoimmunity in NZM2410 mice, but did not observe significant nephritis or proteinuria in either strain. Instead, both treated strains developed splenomegaly with macrophage expansion, cytopenias, and early death consistent with histiocytosis, possibly histiocytic sarcoma (spleen pathology revealed loss of normal tissue architecture). An alternative etiology is an MAS-like syndrome leading to overwhelming inflammation, since others have demonstrated MAS-like disease with repetitive TLR stimulation (TLR2,4,9) (26–29). More detailed studies would be needed to determine whether MAS-like pathogenesis is playing a role in this model. For investigators who are focused on murine models of lupus nephritis, this model is not ideal for use in B6 mice, however topical TLR7 agonists may be useful as inducers or accelerants of autoimmunity and other lupus-like manifestations. It may be particularly relevant for those studying development of inflammatory hemophagocytes secondary to autoimmune conditions such as lupus.

## Data Availability Statement

The datasets generated for this study are available on request to the corresponding author.

## Ethics Statement

The animal study was reviewed and approved by Ralph H. Johnson Department of Veterans Affairs Medical Center Institutional Animal Care and Use Committee (VA IACUC).

## Author Contributions

MC directed the work, contributed to designing the study, and reviewed/interpreted the data. MC and JW conducted all experiments with help from IM (ELISAs, animal breeding and genotyping), PR (renal pathology), and SC-O (spleen pathology and spleen IHC). MC and JW prepared the manuscript and figures. All authors read and approved the final manuscript.

### Conflict of Interest

The authors declare that the research was conducted in the absence of any commercial or financial relationships that could be construed as a potential conflict of interest.
