# Supplementary figures and images for: TLR7 Agonism Accelerates Disease and Causes a Fatal Myeloproliferative Disorder in NZM 2410 Lupus Mice
Source: Front Immunol. 2020 Jan 10;10:3054. doi: 10.3389/fimmu.2019.03054 (PMC6967132; doi:10.3389/fimmu.2019.03054)

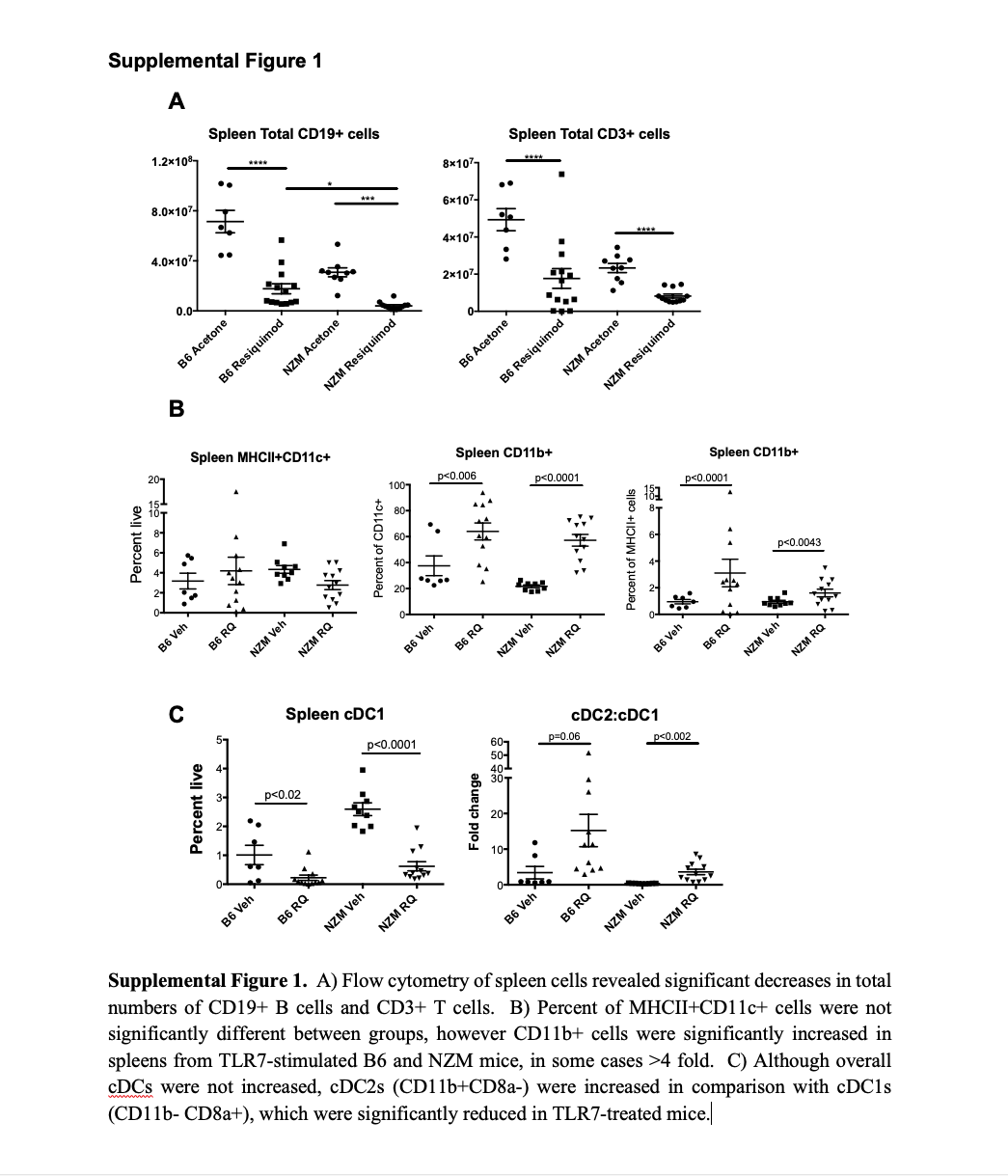

Supplement: Supplementary file 1 [file Image_1.TIFF]

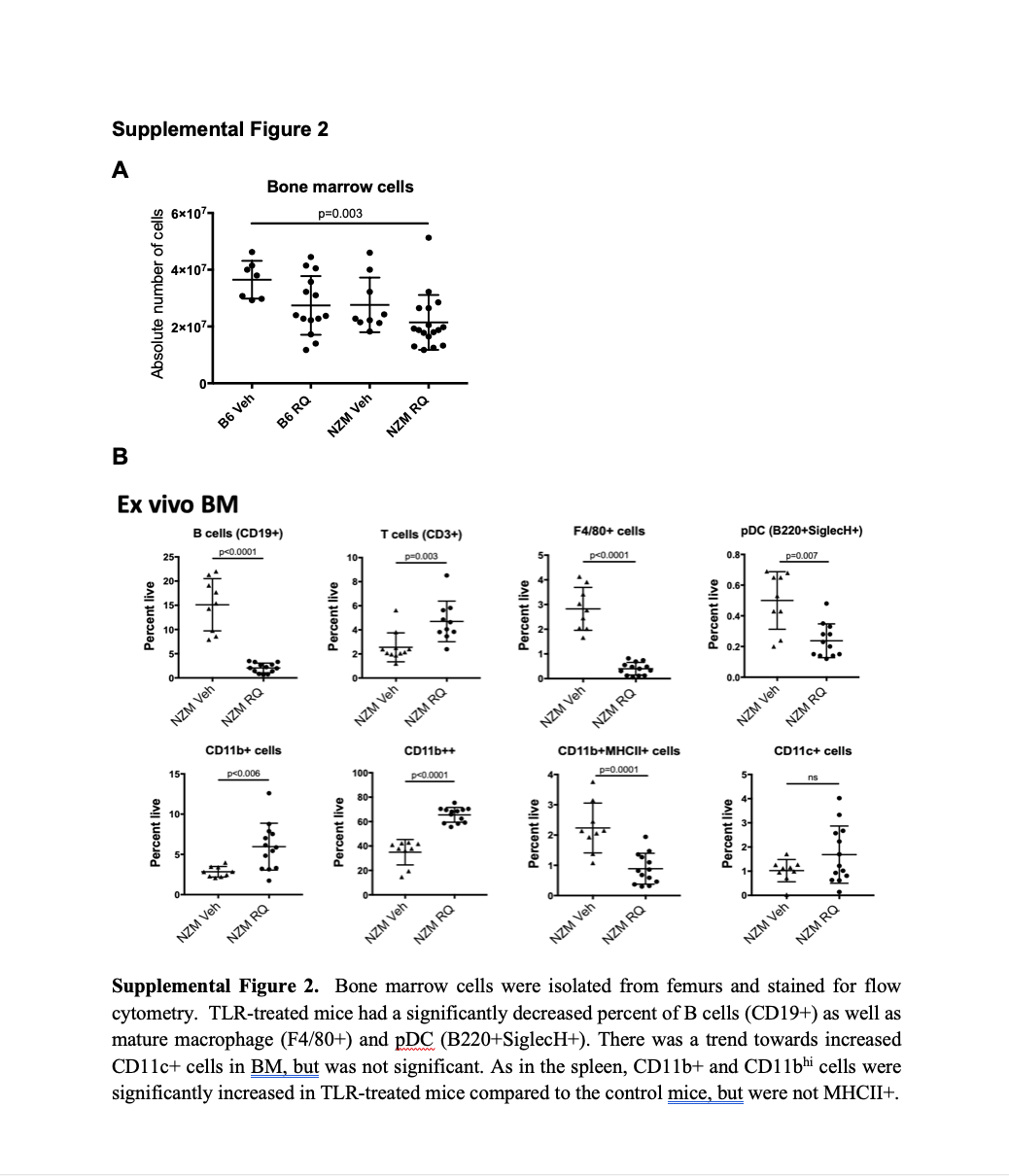

Supplement: Supplementary file 2 [file Image_2.TIFF]
